# Integrative method to detect invasive mealybug (Hemiptera, Coccomorpha, Pseudococcidae) species on highways in Hungary: evidence for northward spread of Comstock mealybug

**DOI:** 10.3897/BDJ.13.e163732

**Published:** 2025-09-10

**Authors:** Éva Szita, Kornél Gerő, Janka Simon, M. Bora Kaydan

**Affiliations:** 1 National Laboratory for Health Security, Plant Protection Institute, HUN-REN Centre for Agricultural Research, Budapest, Hungary National Laboratory for Health Security, Plant Protection Institute, HUN-REN Centre for Agricultural Research Budapest Hungary; 2 Biotechnology Application and Research Centre, Çukurova University, Balcalı, Adana, Turkiye Biotechnology Application and Research Centre, Çukurova University Balcalı, Adana Turkiye

**Keywords:** invasive alien species, molecular tools, pheromone trap, *Pseudococcus comstocki*, scale insect

## Abstract

Climate change, coupled with the intensification of road transport and global trade, has contributed to a significant increase in the number of newly introduced scale insect species into new regions. Invasive alien pests represent a significant threat to agriculture and forestry, resulting in considerable economic losses globally. In this study, three potentially invasive mealybug species (Hemiptera, Coccomorpha, Pseudococcidae): *Pseudococcus comstocki* (Kuwana), *Planococcus citri* (Risso) and *Planococcus ficus* (Signoret) were monitored using a pheromone trap network along highways in Hungary during 2020–2021. Prior to this survey, none of these species had been detected under open-field conditions in Hungary. Specimens captured were identified through analysis of cytochrome oxidase I (COI) gene fragments. The presence of *Ps. comstocki* was confirmed for the first time in outdoor environments at six localities in Hungary using both pheromone trapping and molecular techniques. It is currently the northernmost documented outdoor occurrence of *Ps. comstocki* in Europe. In contrast, *Pl. citri* and *Pl. ficus* were not detected in open-air conditions during the survey.

## Introduction

Climate change, combined with the intensification of road transport and global trade, has led to a significant increase in the number of newly introduced arthropod species worldwide (Mazzeo et al. 2014, Haris et al. 2025). Agriculture, horticulture and forestry are directly affected by the expansion of the distribution area of several invasive pest species (Kellomäki et al. 2000, Dawson et al. 2011, Haraszthy 2022). New invasive insect pests are emerging in various environments, including fruit orchards, vineyards, in urban landscapes on ornamental plants, as well as in forestry (Csóka 1997, Melika et al. 2003, Fetykó et al. 2012, Kozár et al. 2013a, Miller et al. 2014, Vétek 2016, Csóka and Hirka 2017, Nagy et al. 2017, Szita and Érsek 2017, Haraszthy 2022, Ciceu et al. 2024). The economic impact of the invasions is extensive, encompassing loss of products, extra expenses in plant protection, challenges related to quarantine and trade regulations and deterioration of green spaces in urban parks due to pest-related nuisances (Miller et al. 2014, Kondo and Watson 2022). In addition, Hungary is amongst the European countries most vulnerable to climate change, with mean temperatures increasing at a rate approximately 1.5 times higher than the continental average, thereby elevating the risk of pest establishment (VAHAVA 2006, Faragó et al. 2010, Policy Solutions 2011, Mezösi et al. 2013, Haris et al. 2025).

Road networks play a significant role in facilitating the spread of various plant and animal species. Small organisms like insects can be passively transported over hundreds of kilometres by vehicles (Haraszthy 2022, Haris et al. 2025). Simultaneously, the green buffer zones along highways often serve as corridors within agricultural landscapes, further aiding species dispersal; for example, the spread of *Diabrotica virgifera* LeConte (Coleoptera, Chrysomelidae) has been traced along major roads from Serbia through France and into Ukraine (Kiss et al. 2005, Fokin 2006). A similar pattern was observed in case of *Cameraria ohridella* Deschka & Dimic (Lepidoptera, Gracillariidae) (Horváth and Benedek 1986, Gilbert et al. 2004) and recent research on the Hungarian highway network has revealed the presence and rapid spread of *Drosophila suzukii* (Matsumura) (Diptera, Drosophilidae) (Kiss et al. 2013, Lengyel et al. 2015). This invasive species caused near-total losses in raspberry and mulberry crops for several growers in northern Hungary in 2016, just four years after its initial detection in the country (Kiss et al. 2017, Orosz et al. 2018).

The study of scale insect (Hemiptera, Coccomorpha) assemblages of highways in Hungary began in the early 2000s (Kozár et al. 2004, Kozár 2009, Kozár et al. 2012). These investigations revealed a remarkably diverse scale insect fauna within the rest areas of Hungarian highway system, identifying 137 species (Kozár et al. 2013b), which accounts approximately half of the known scale insect species in Hungary (Kozár et al. 2013a). In addition to establishing a foundational understanding of the scaleinsect fauna associated with Hungarian road networks, particular emphasis was placed on the detection of potentially invasive mealybug (Pseudococcidae) species, as their suitability for passive long distance dispersal poses a significant risk of range expansion and consequent ecological and economic impacts in newly colonised areas (Tóbiás et al. 2010, Kondo and Watson 2022).

Mealybugs represent the second largest family of scale insects, comprising 264 genera and 2065 species (García Morales et al. 2016), some of which are significant pests in agriculture and horticulture (Kondo and Watson 2022). These small, sap-sucking insects are covered with a fine powdery wax and often possess lateral wax filaments around their entire body or are restricted to the posterior segments only (Williams 2004, Kondo and Watson 2022). Adult females are neotenic and wingless, with body sizes ranging from 0.4 to 5.0 mm, depending on the species (Kosztarab and Kozár 1988, Williams and Granara de Willink 1992). Adult females either lay eggs into a white, filamentous waxy ovisac secreted from glands in the cuticle or are ovoviviparous, in which case they generally lack an ovisac (Ferris 1950, McKenzie 1967). Female development includes three immature instars, while males have four instars (Kosztarab and Kozár 1988, Danzig and Gavrilov-Zimin 2014). Adult males, if present, are non-feeding and short-lived, making them rarely collected (Kosztarab and Kozár 1988, Williams 2004).

Three species of potentially invasive mealybug pests were selected for investigation at highway rest areas in Hungary, namely *Planococcus citri* (Risso), *Planococcus ficus* (Signoret) and *Pseudococcus comstocki* (Kuwana). These species are key scaleinsect pests in vineyards and orchards in Mediterranean countries; all three have shown a substantial northward spread in different parts of Europe, especially in the last forty years (Kozár and Nagyné Dávid 1986, Kozár 1997, Kozár 1998, Godinho and Franco 2001, Kozár et al. 2004, Masten Milek and Simala 2008, Karamaouna et al. 2010, Tena and Garcia-Marí 2011, Pellizzari et al. 2012, Pacheco da Silva et al. 2014, Pellizzari and Porcelli 2014, García Morales et al. 2016, Malausa et al. 2016, Mansour et al. 2017). *Planococcus citri* had been detected in Hungary on several occasions on imported ornamental plants and tropical fruits; however, it has no established outdoor populations (Kozár and Szentkirályi 2005, Fetykó et al. 2012, Kozár et al. 2013a). In contrast, *Ps. comstocki* and *Pl. ficus* have not been recorded in Hungary to date (Tóbiás et al. 2010, Kozár et al. 2013a, Kondo and Watson 2022).

Although *Pl. citri*, *Pl. ficus* and *Pseudococcus comstocki* were not detected living outdoors in Hungary during the 2009–2013 survey (Tóbiás et al. 2010, Kozár et al. 2013a), their potential to establish in the country remains high. The present study aimed to re-initiate the investigation of these invasive mealybug species in Hungary, using integrative methodology combining molecular diagnostic tools and pheromone trapping on highways, to enable the early detection of any of these three mealybug pests in Hungary.

## Material and methods

### Pheromone trap network

**Figure 1.**
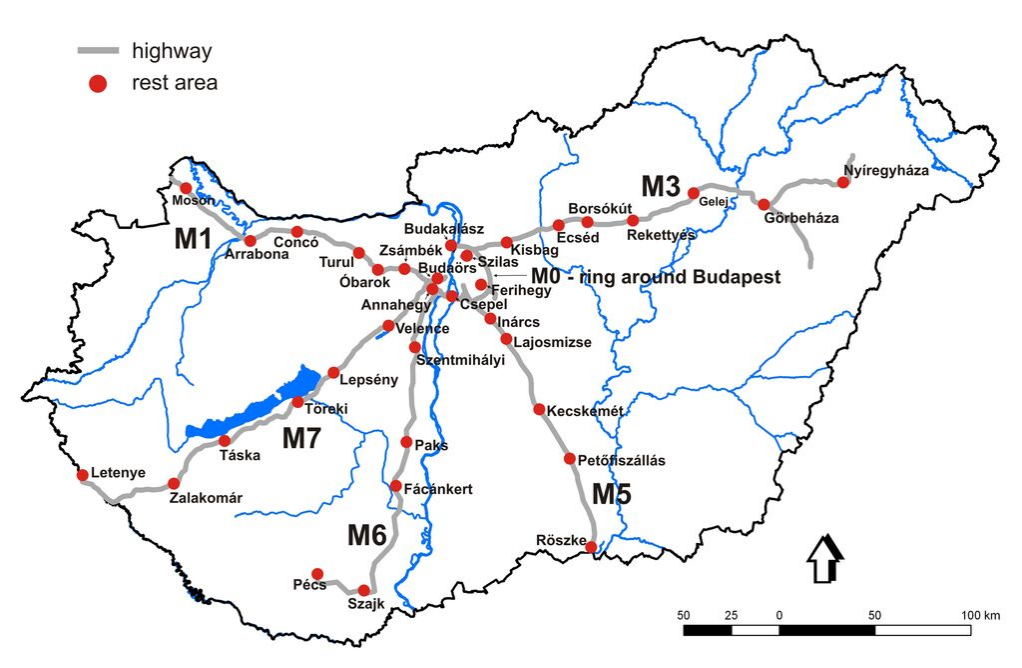
Pheromone-trap sample sites on highways in Hungary.

**Figure 2.**
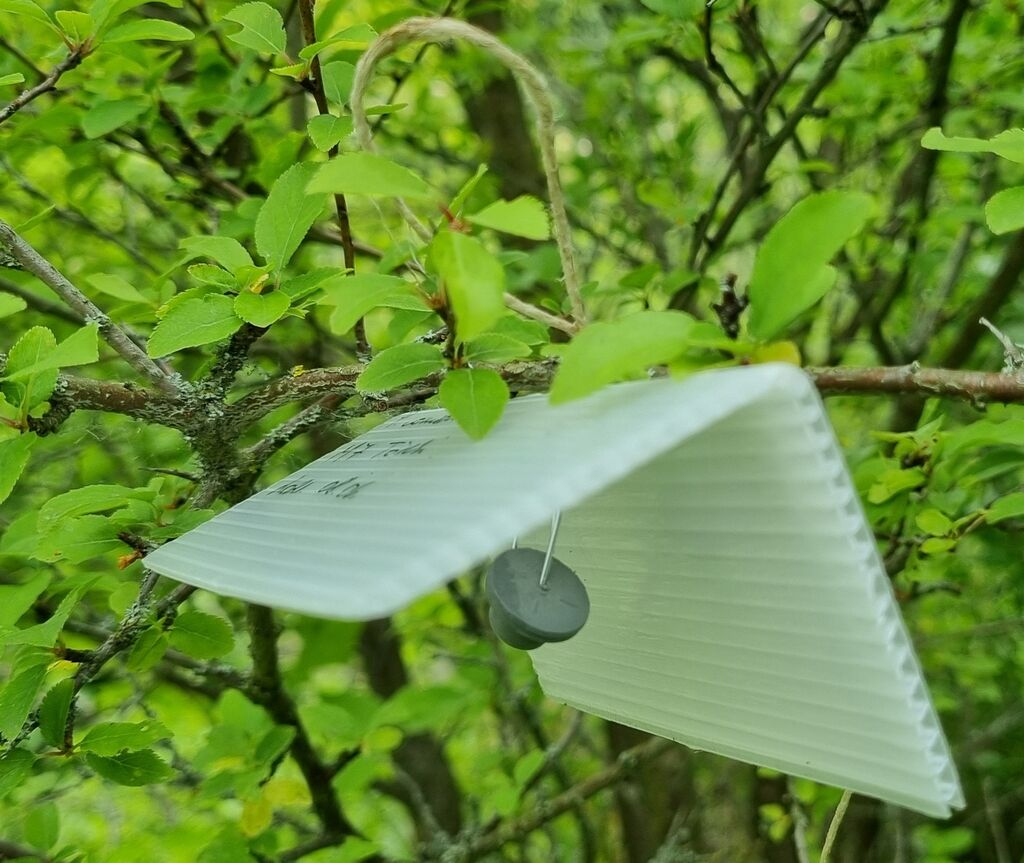
The type of semi-transparent plastic tent traps used on highways in Hungary.

A pheromone trap network targeting three potentially invasive mealybug species (*Pl. citri*, *Pl. ficus* and *Ps. comstocki*) was established on the Hungarian highway system across the country, encompassing 35 highway rest areas (Fig. 1). Trapping was conducted between 2020 and 2021, with three trapping periods per season. An additional sampling site was incorporated into the network at the Botanical Garden of University of Pécs (BGUP). The initial traps were deployed during the first ten days of June and subsequently replaced with new traps every four weeks in July and August in both experimental years. Semi-transparent plastic tent traps (10 × 10 cm) with sticky surfaces were used in combination with species-specific pheromone lures to attract male mealybugs (Fig. 2). The pheromone compounds employed were: (+)-2,2-dimethyl-3-(1-methylethenyl) cyclobutanemethanol acetate for *Planococcus citri*; (S)-lavandulyl senecioate and (S)-lavandulyl isovalerate for *Pl. ficus*; and 2,6-dimethyl-1,5-heptadien-3-ol acetate for *Pseudococcus comstocki*. Pheromone lures were sourced from Biochemtech Ltd. (Chișinău, Moldova). Soveurode spray glue (Witasek Pflanzenschutz GmbH, Austria) was used as an adhesive in the tent traps, which preserved the DNA of captured insects for subsequent molecular analyses (Tóbiás et al. 2010).

Pheromone traps provide an effective and straightforward method for investigating the dispersal of scale insect pests; however, they are limited to capturing the winged males. Successful application of this technique requires fundamental knowledge of male morphology, yet the identification of male scale insects remains problematic, as existing taxonomic descriptions and keys are predominantly based on characteristics of the females (Kosztarab and Kozár 1988, Gill 1997). Furthermore, pheromone lures are not always completely species-specific, occasionally resulting in the capture of non-target species or insects inadvertently trapped by wind-driven movement (Frey and Frey 1995, Kozár et al. 1996, Kozár et al. 1997). Therefore, molecular diagnostic tools are essential to reliably confirm the identity of captured males, particularly when the number of specimens caught are low.

### DNA extraction, amplification and sequencing

Prior to DNA extraction, all specimens were examined under a stereomicroscope (Olympus SZ40) for visually detectable fungal infection. DNA was extracted from individual fungal-infection-free adult males using the Qiagen DNA-easy Tissue Kit (Qiagen, Inc, Valencia, CA). The mealybugs were not crushed before extraction; instead, the cell lysis time was extended beyond the manufacturer’s recommendations (Malausa et al. 2009).

PCR products were generated from a mitochondrial gene, cytochrome oxidase I part 2 (COI). Primers for both amplification and sequencing were 5’–CAACATTTATTTTGATTTTTTGG–3’ (C1-J-2183 aka Jerry) and 5’–GCWACWACRTAATAKGTATCATG–3’ (C1-N-2568 aka BEN3R; designed by T.R. Schultz, Smithsonian Institution) (Gullan et al. 2003).

PCR reaction components and final concentrations were 1.5–2.5 mM MgCl_2_, 0.2 mM dNTPs and 1 unit of *Taq* polymerase in a proprietary buffer (PCR Master Mix, Promega Corporation, USA), 0.5 μM of each primer and 3.6 μl DNA template in a final volume of 25 μl. The PCR cycling protocol for COI was 95°C for 7 mins, followed by 40 cycles of 95°C for 1 min, 45°C for 1 min and 72°C for 1 min 30 s, with a final extension at 72°C for 4 mins (Kaydan et al. 2023). PCR products were purified using NucleoSpin® Gel and PCR Clean-up (Macherey and Nagel) according to the manufacturer’s instructions and were sequenced on both strands by Macrogene Europe (Amsterdam, The Netherlands).

Contigs were assembled using CodonCode Aligner v. 10.0.2 (CodonCode Corporation). Multiple sequence alignments were performed by using ClustalW implemented in BioEdit v. 7.2 (Informer technologies, Inc.). All alignments were manually controlled and trimmed to remove regions containing large gaps, ensuring alignment quality. The consensus sequence for each specimens was queried against the GenBank database using the NCBI Nucleotice BLAST tool (Camacho et al. 2009) to identify homologous sequences. All resulting sequences have been deposited in the NCBI GenBank under the following accession numbers: PV959471-PV959478.

### Phylogenetic analysis

In order to carry out a complete phylogenetic analysis, DNA sequences from females of other Pseudococcidae species were obtained from public databases and Kaydan et al. (2023); details of the specimens used are shown in Table 1.

Bayesian methods were employed to infer phylogenetic trees using MrBayes version 3.2.6 (Huelsenbeck and Ronquist 2001), integrated into the Geneious Prime version 2025.1.2 software package. Each data partition was assigned a separate GTR model with gamma-distributed rates and a proportion of invariant sites, applying default priors. Four Markov chains, three hot and one cold, were run concurrently for ten million generations, with trees sampled every 1000 generations. The log-likelihood over time was monitored and the first 1000 trees, generated prior to reaching stationarity, were discarded as burn-in. Consensus tree was built using Geneious Tree Builder panel in Geneious Prime 2025.1.2 software package using the Neighbour-Joining method and HKY as the genetic distance model, performing ten thousand non-parametric bootstrap replicates.

## Results

In the 2020 sampling season, no male pseudococcid specimens were captured along the highway network, except in the *Planococcus citri* pheromone traps placed at BGUP, where a severe infestation of *Pl. citri* was recorded in the nearby greenhouses.

In 2021, mealybug males were captured in 15 pheromone traps and eight of them yielded usable DNA (Table 2). The presence of *Pseudococcus comstocki* was confirmed in seven traps from six localities. Although two of these traps were baited with *Pl. citri* lures, COI sequence data verified that the specimens collected were *Ps. comstocki*. Currently, based on male captures, the population density appears to be too low to enable the detection of female specimens. *Pl. citri* was detected at a single locality, BGUP; however, this is not considered a true outdoor capture, as the trap was located in close proximity to the BGUP greenhouse that was known to be infested with *Pl. citri*. *Pl. ficus* was not detected during the survey.

### Phylogenetic analysis

Fragments of the COI gene, 179–389 base pairs in length, were amplified from each sample and used for phylogenetic analysis.

**Figure 3.**
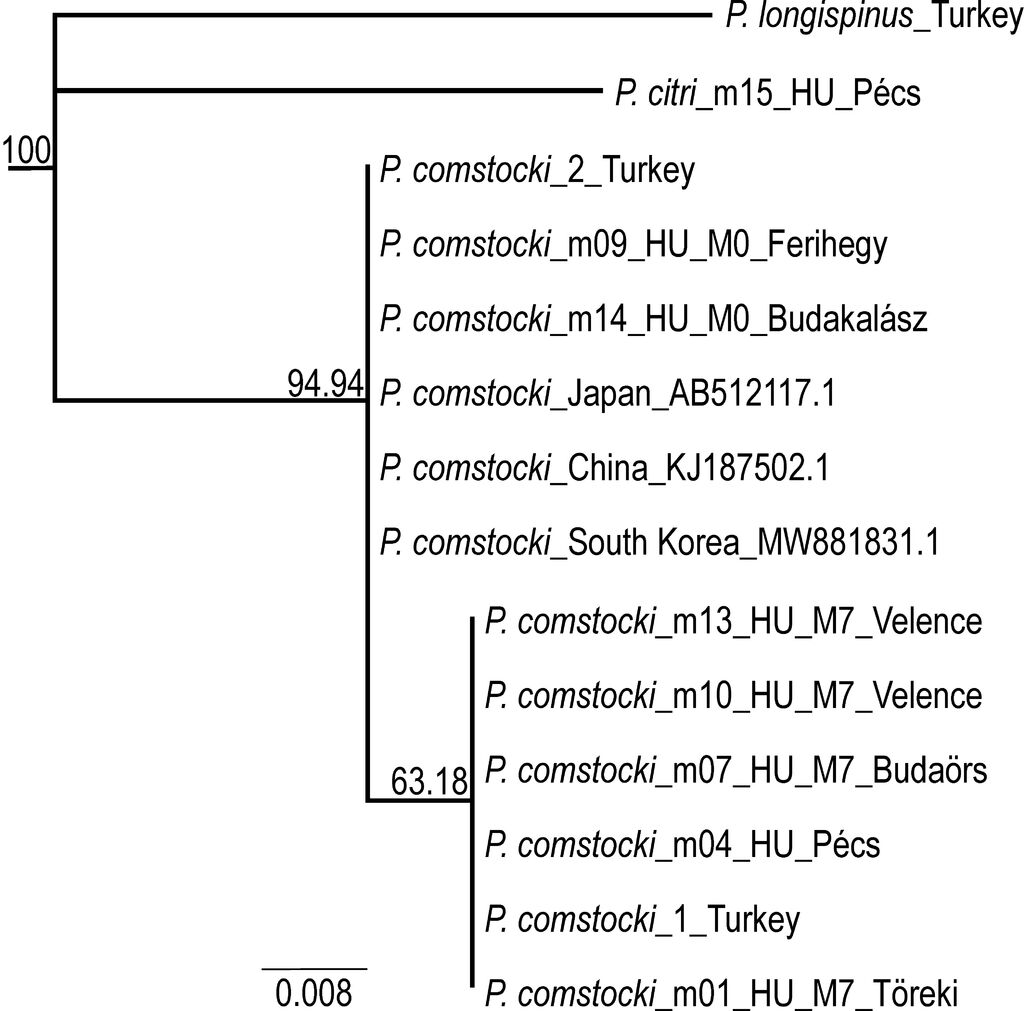
Consensus tree of examined pseudococcid samples, based on COI gene fragments.

COI sequences from male specimens were combined with previously published *Pseudococcus comstocki* sequences from the NCBI GenBank database and Kaydan et al. (2023) to verify species identity (Fig. 3). The resulting phylogenetic tree reveals two distinct haplotypes of *P. comstocki* based on the COI region. Despite the clustering of these haplotypes into separate clades, the low degree of genetic divergence between them, along with the weak support value for the corresponding node, indicates that both haplotypes belong to the same species.

## Discussion

The occurrence of the invasive mealybug species, *Ps. comstocki* (Comstock mealybug) was confirmed under open-field conditions for the first time in Hungary, using sex-pheromone traps and molecular methods. This currently represents the northernmost outdoor distribution data of *Ps. comstocki* in Europe.

The Comstock mealybug is native to East Asia, but has been accidentally introduced into both America and Europe (Williams and Granara de Willink 1992, Kondo and Watson 2022). Its first recorded occurrences in the Mediterranean region of Europe were reported simultaneously from southern France and northern Italy in 2004 (Kreiter and Germain 2005, Pellizzari 2005). Since then, Comstock mealybug has caused severe economic damage to apple, pear and peach orchards in these regions (Germain 2007, Visigalli et al. 2008, Guerrieri and Pellizzari 2009, Masi et al. 2010, Groussier-Bout et al. 2011, Pellizzari et al. 2012, Malausa et al. 2016, Marchesini et al. 2018, Kreiter et al. 2020). The species has continued to expand its range: it was intercepted in Croatia in 2007 (Masten Milek et al. 2009, Masten Milek et al. 2016), detections have been reported from Turkey since 2005 (Kaydan and Kozár 2011, Ülgentürk et al. 2022), from Greece in 2013 (Szita et al. 2017), from Switzerland since 2016 (Terrettaz et al. 2020, Schneider et al. 2023) and the current study reveals the northerrnmost European record of this species from Hungary.

Although Comstock mealybug was removed from the EPPO plant quarantine list in 1988, it is still regarded as quarantine pest and remains listed on A1 or A2 quarantine lists in a few European countries (Azerbaijan, Belarus, Georgia), as well as in regions on other continents (e.g. Egypt, Morocco, Chile, Mexico, Israel, Kazakhstan, Uzbekistan) (EPPO 2016, EPPO 2019, EPPO 2024, EPPO 2025).

*Pseudococcus comstocki* is highly polyphagous, feeding on plant species in 81 genera belonging to 48 families (García Morales et al. 2016). Its primary hosts include a variety of economically important fruit crops such as apple (*Malus*), peach (*Prunus*), pear (*Pyrus*) and grapevine (*Vitis vinifera*), as well as several ornamental plants commonly found in urban environments (e.g. *Catalpa*, *Prunus laurocerasus*, *Morus* spp.) (García Morales et al. 2016, Kondo and Watson 2022, EPPO 2025). Direct damage is caused by the insect’s phloem-feeding activity, which leads to fruit spotting, altered fruit texture and the formation of knot-like galls on the bark and along leaf veins (Pellizzari et al. 2012, Kondo and Watson 2022). Indirect damage results from the accumulation of sugary honeydew waste on plant surfaces, providing a suitable substrate for the growth of black sooty moulds, so reducing photosynthetic efficiency of the plants and sometimes leading to early defoliation. Additionally, Comstock mealybug is considered a vector of plant virus diseases, further exacerbating its impact on agricultural and ornamental hosts (Nakaune et al. 2008, Malausa et al. 2016, Herrbach et al. 2017).

Comstock mealybug overwinters in the egg stage and has 2–4 generations per year, depending on climatic conditions (Pellizzari et al. 2012, Kondo and Watson 2022). Pellizzari et al. (2012) enumerates several countries with different numbers of generation of Comstock mealybug worldwide, with development of two generations per year in Ukraine (Odessa Region) (Romanchenko and Bel'skaya 1981) and in USA (New York State) (Agnello et al. 1992), three generations annually in Italy (Pellizzari et al. 2012) and USA (Virginia) (Hough 1925, Kosztarab 1996) and 3–4 generations annually in USA (California) (Meyerdirk and Newell 1979). Based on the weather patterns of the above-mentioned territories, two generations per year may be expected in Hungary.

## Conclusions

The appearance of *Ps. comstocki* in Hungary was expected (Kozár 2009, Tóbiás et al. 2010); however, its simultaneous detection at six geographically distinct locations suggests that natural dispersal is an unlikely explanation for its establishment in Hungary. Given that all the infested highway rest areas are situated within highly urbanised areas and that plant replacement is quite common at highway rest stops, we hypothesise that *Ps. comstocki* was most probably introduced via infested plant material. To date, no female specimens have been detected, presumably due to the low population density. Nevertheless, the confirmed establishment of this species outdoors and its potential for further spread pose a significant risk to both commercial fruit production and urban environments in Hungary.

Our findings demonstrate that the integration of sex-pheromone trapping and molecular diagnostic tools offers an effective approach for the early detection of invasive pest species. Although current population levels appear to be low, further research is needed to assess the dispersal of the Comstock mealybug. Therefore, continued monitoring of *Ps. comstocki* as well as *Planococcus citri* and *Pl. ficus* is strongly recommended.

## Data resources

NCBI GenBank

## Figures and Tables

**Table 1.** List of external sources of sequences used in the molecular data analysis.

**Species**	**Sex**	**Accession no.**	**Country**	**Source**
*Pseudococcus comstocki*	female	NCBI AB512117.1	Japan	Unpublished (Toda S., Narai Y. and Sawamura N.)
*Pseudococcus comstocki*	female	NCBI KJ187502.1	China	Unpublished (Wu Z.F.)
*Pseudococcus comstocki*	female	NCBI MW881831.1	South Korea	(Choi and Lee 2022)
*Pseudococcus comstocki*	female	MBK148	Türkiye	(Kaydan et al. 2023)
*Pseudococcus comstocki*	female	MBK213	Türkiye	(Kaydan et al. 2023)
*Pseudococcus longispinus*	female	P.long_2_TR	Türkiye	(Kaydan et al. 2023)

**Table 2.** Collection data of male *Planococcus citri* and *Pseudococcus comstocki* from pheromone traps, which provided usable DNA.

**DNA sample**	**Pheromone lure**	**NCBI matching**	**Location**	**Coordinates**	**Collection period**
M15	*Pl. citri*	*Pl. citri*	BGUP	46°4'39.74"N 18°12'20.33"E	07.07–08.11.2021
M04	*Ps. comstocki*	*Ps. comstocki*	BGUP	46° 4'39.74"N 18°12'20.33"E	08.11–10.05.2021
M14	*Pl. citri*	*Ps. comstocki*	M0 Budakalász	47°36'36.41"N 19°3'42.61"E	08.11–10.05.2021
M09	*Ps. comstocki*	*Ps. comstocki*	M0 Ferihegy	47°25'12.24"N 19°15'15.60"E	08.11–10.05.2021
M07	*Ps. comstocki*	*Ps. comstocki*	M7 Budaörs	47°27'6.81"N 18°57'46.28"E	08.06–10.08.2021
M01	*Ps. comstocki*	*Ps. comstocki*	M7 Töreki	46°52'59.76"N 18°0'43.72"E	08.06–10.08.2021
M10	*Pl. citri*	*Ps. comstocki*	M7 Velence	47°14'34.87"N 18°38'0.76"E	08.06–10.08.2021
M13	*Ps. comstocki*	*Ps. comstocki*	M7 Velence	47°14'34.87"N 18°38'0.76"E	08.06–10.08.2021
